# Current Challenges in Breast Implantation

**DOI:** 10.3390/medicina57111214

**Published:** 2021-11-07

**Authors:** Zuzanna Pelc, Magdalena Skórzewska, Andrzej Kurylcio, Paweł Olko, Joanna Dryka, Piotr Machowiec, Marcela Maksymowicz, Karol Rawicz-Pruszyński, Wojciech Polkowski

**Affiliations:** Department of Surgical Oncology, Medical University of Lublin, Radziwiłłowska 13 St., 20-080 Lublin, Poland; zuzanna.torun@gmail.com (Z.P.); magdalena.skorzewska@umlub.pl (M.S.); andrzej.kurylcio@umlub.pl (A.K.); pawel11.olko@gmail.com (P.O.); asia.dryka@wp.pl (J.D.); piotr.machowiec1997@gmail.com (P.M.); marcela.maksymowicz@gmail.com (M.M.); wojciech.polkowski@umlub.pl (W.P.)

**Keywords:** breast implantation, breast implant illness, breast implant-associated anaplastic large cell lymphoma

## Abstract

Breast implantation (BI) is the most common plastic surgery worldwide performed among women. Generally, BI is performed both in aesthetic and oncoplastic procedures. Recently, the prevalence of breast implant-associated anaplastic large cell lymphoma (BIA-ALCL) or breast implant illness (BII) has aroused concerns. As a result, several countries, like Australia, Korea or the United Kingdom, introduced national registries dedicated to the safety and quality of BI surgeries. This narrative review aimed to focus on the clinical challenges, management and the current state of knowledge of BI. Both short and long-term outcomes of BI are determined by various alternatives and differences, which surgeons must consider during the planning and performing breast augmentation along with further complications or risk of reoperation. Proper preoperative decisions and aspects of surgical technique emerged to be equally important. The number of performed breast reconstructions is increasing, providing the finest aesthetic results and improving patient’s quality of life. Choice of prosthesis varies according to individual preferences and anatomical variables. A newly diagnosed cases of BIA-ALCL with lacking data on prevention, diagnosis, and treatment are placing it as a compelling medical challenge. Similarly, BII remains one of the most controversial subjects in reconstructive breast surgery due to unspecified diagnostic procedures, and recommendations.

## 1. Introduction

Breast implantation (BI) is the most common plastic surgery worldwide performed among women [1]. In 2019, nearly 2 million breast augmentation surgeries were performed, comprising 15.8% of all plastic surgery procedures [1]. In 2020, in the United States, there were more than 300,000 procedures involving breast augmentation and reconstruction surgeries [2]. Breast augmentation was performed among 58.4% of patients, mainly aged 30 to 40 [2]. Compared with the data from 2000, the number of breast augmentation surgeries increased by 41%, and its demand pursues to enhance annually [2,3]. Generally, BI is performed both in aesthetic and oncoplastic procedures. Prophylactic or curative mastectomy followed by reconstruction surgery reestablishes the breast’s primary size, contour, and consistency. Since the 19th century, different types of BI have evolved from simple fat transfer to advanced implants providing the most satisfactory aesthetic results and improving patient’s quality of life. Choice of prosthesis varies according to individual preferences, anatomy, tissues elasticity and surgeon’s experience. However, with progress being made, new complications have emerged. Recently, the prevalence of breast implant-associated anaplastic large cell lymphoma (BIA-ALCL) or breast implant illness (BII) has aroused concerns. As a result, several countries, including Australia, Korea or the United Kingdom, introduced national registries dedicated to the safety and quality of BI surgeries [4]. This narrative review aimed to focus on the clinical challenges, management and the current state of knowledge of BI.

## 2. Materials and Methods

**Figure 1:**
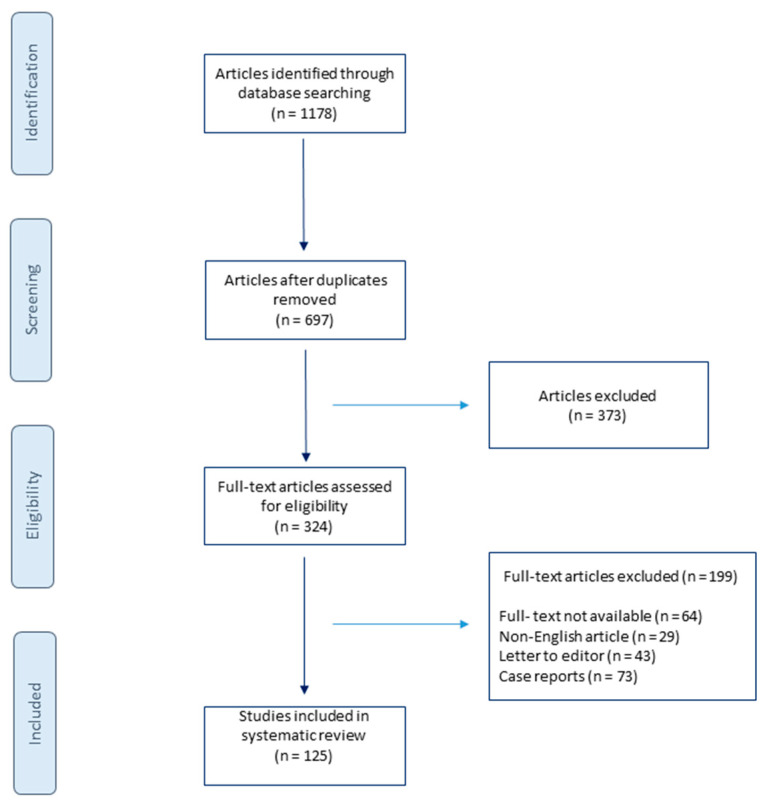
Flowchart of the literature search.

A comprehensive literature search using the MEDLINE, EMBASE, and Scopus database was performed on the 16 June 2021 and updated on the 21 October 2021. The search strategy included the following keywords and medical subject heading (MeSH) terms: “breast implantation” OR “breast implant” OR “breast augmentation” OR “breast implant illness” OR “BII” OR “breast implant anaplastic-large cell lymphoma” OR “BIA-ACL” OR “breast implant removal” OR “breast implant rupture” OR “breast implant rupture”. The search was limited to articles published in English. The studies included in this review were based on the eligibility criteria. This review’s inclusion criteria were as follows: only English language, full texts articles, published since 1993, describing the population over the age of 18. Studies were excluded if the abstract revealed no relevance to the subject or if they were one of the following: case reports, letters, editorials, animal studies. For publications without an abstract, the full text was retrieved and assessed for eligibility. If the full text was not available, the report was excluded. After the extraction of the studies based on the eligibility criteria, the articles were screened independently by two investigators (ZP and KRP). First, studies were screened based on title and abstract, followed by eligibility screening. Detailed search results are shown in the flow diagram in Figure 1.

## 3. Step Back in Time

The first breast augmentation was performed at the end of the 19th century. In 1895, Vincenz Czerny, an Austrian-German surgeon, applied adipose tissue obtained from a lipoma of the lumbar region to compensate for the asymmetry of the breast after partial mastectomy [5,6]. In 1904, ineffective attempts to use paraffin injections from a combination of petroleum jelly and olive oil have been made [5]. Due to the substantial body’s reaction to a foreign body, hard masses, inflammation, or even extensive tissue necrosis were formed. Complications of this procedure also included pulmonary embolism or blindness caused by embolism of the cerebral arteries [5]. The unsuccessful paraffin injections initiated the research for other methods of effective breast augmentation. The attempt to use liquid silicone injections was introduced, but similarly, the substance was poorly tolerated and caused many side effects including death. Food and Drug Administration (FDA) never endorsed this procedure, therefore the search for the appropriate material continued. Numerous unsuccessful trials to use polyvinyl alcohol sponges and polyethene tapes proved no positive results [7]. Moreover, these substances presented many adverse effects as local tissue reactions, deformation of the breasts and discomfort [5]. In 1962, Cronin and Gerow developed implants with silicone gel filling and a synthetic shell [8,9], leading to a historic breakthrough in the field of breast augmentation [10]. In 1964, the first saline-filled breast implants were developed and produced by the French company Laboratoires Arion [9].

## 4. Silicone Breast Implants Crisis

In the early 1990s [11], first reports about the potential link between silicone breast implants and autoimmune or rheumatic diseases led to doubts concerning the safety of silicone implants. As a result, the FDA precluded third-generation silicone implants [6,12] without any specific reason [13]. Consequently, silicone breast implants were unapproachable [13]. For two years saline-filled implants were the only method accepted by the FDA for aesthetic breast augmentation. Additionally, the consensus was limited to a narrow group of procedures: temporary expanders awaiting for permanent reconstructive surgery, reconstructive surgery during mastectomy, and ruptured silicone gel breast implants pending replacement [5,8]. The decision of the FDA to suspend the use of silicone implants and requesting manufacturers to provide additional data on both the safety and effectiveness of the 3rd generation silicone implants resulted in the creation of the 4th and 5th generation silicone implants [6,12]. These implants have been designed following the stringent guidelines of the American Society for Testing Methodology (ASTM) and the FDA [6,12]. New generated implants were characterized with better quality and a more comprehensive range of surface textures and shapes due to new parameters of the implant shell’s thickness and the cohesiveness of the gel filling.

**Figure 2:**
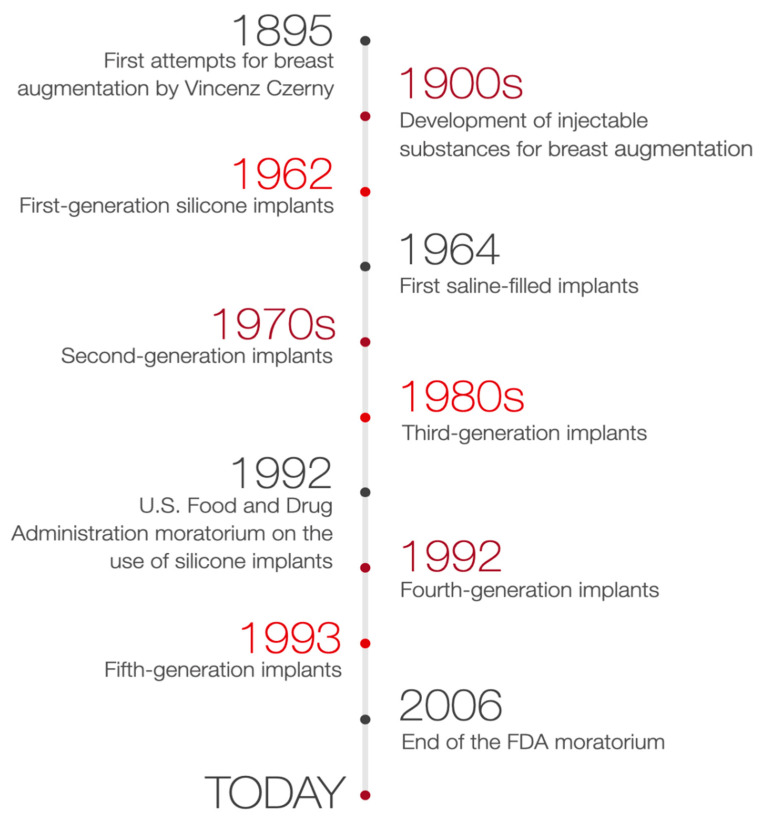
Timeline of breast augmentation history.

In consequence, after 14 years of suspension, silicone breast implants were reintroduced in the United States [9]. The decision of the FDA was supported by many studies proving silicone breast implants safety and its role in improving the quality of patients’ life. Therefore, with the benefit of hindsight, it is clear that the suspension of silicone breast implants has brought many advantages. As a result, a few studies have been performed to verify the effects of silicone implants rupture, their relationship to autoimmune diseases, or patient satisfaction after breast augmentation surgery [9,14]. Silicone implants of the 4th and 5th generation, created due to the withdrawal of 3rd generation silicone implants, together with saline-filled implants, are currently used [10]. The timeline of breast augmentation history is shown in Figure 2.

## 5. Characteristics of Breast Implants

Breast implants are applied in plastic surgery for breast augmentation or reconstruction surgery among patients undergoing a partial or complete mastectomy due to oncological or aesthetic reasons [15]. There are two primary breast implants: saline- and silicone gel-filled implants [16,17,18] composed of a silicone elastomer shell with a smooth or textured surface [16,17]. Due to the controversy surrounding silicone breast implants and the suspension of their use, between 1992–2006, saline implants were popular mainly in the U [18]. In other parts of the globe, silicone implants were considered superior and more durable, so they remained more common, and this tendency continues to this day. Even in the United States, silicone gel-filled implants are used in approximately 60% of cases [19]. Saline implants are currently regarded as the second-choice option due to problems with deflation, underfilling or overfilling [20]. The water-like consistency, which does not resemble normal breast tissue during palpation, is also a disadvantage that does not apply to silicone implants [18]. Nonetheless, saline implants present undeniable superiorities allowing for a significantly smaller incision or insertion and inflation in situ of an uninflated implant [19]. There is also no need for special long-term monitoring of the results. Considering implants surfaces, fillings with a smooth structure are mainly used in the USA (87% versus 13%) [12]. Nevertheless, in Europe and Australia, implants with a textured surface are applied in almost 90% of cases. Implants differ in their chemical composition and texture and occur in highly diverse and varied sizes and shapes [18]. The form of breast implants can be round or anatomical. To allow a better individual adaptation to the patient, most of the implants characterized as round are available in different projections of a given volume. However, a natural asymmetric shape of anatomical implants provides a broader choice of dimensions. 

**Figure 3:**
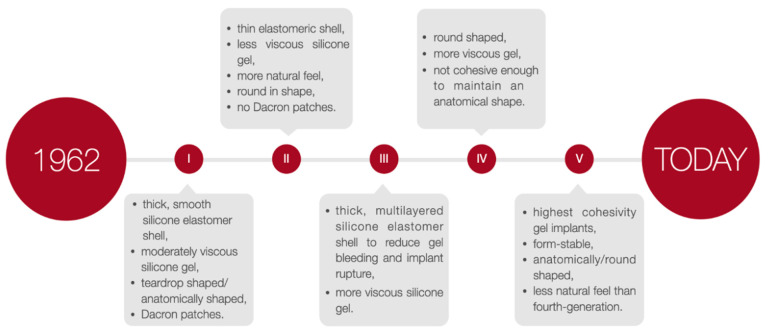
The generations of silicone breast implants.

To select the implant most suited to the patient’s needs, the implants height, width, and projection can be adjusted. Nevertheless, it is recommended that surgeons operate with both round and anatomical implants and adapt the specific type of implant individually. The choice of a particular type of implant may vary depending on the situation. According to the FDA guidelines, a patient must be at least 18 years old to undergo saline-filled breast implant surgery and 22 years old to be qualified for silicone gel-filled implants [15]. The generations of silicone breast implants are shown in Figure 3.

## 6. Breast Augmentation Surgery—Preoperative Management

Breast augmentation surgery for 45 years was considered an isolated surgical procedure, consisting of a breast implant placement in a pocket created by a plastic surgeon. The procedure has been redefined and extended beyond the actual placement of a breast implant [18]. It occurred that breast augmentation surgery is more complicated than initially thought. Both short and long-term outcomes of the procedure are determined by various alternatives and differences, which surgeons must consider during the planning and performing breast augmentation surgeries and further complications or risk of reoperation [21]. Therefore, proper preoperative decisions and aspects of surgical technique emerged to be equally important. One of the controversies is the manner of one or two-stage surgery [22]. Traditional and safe two-stage reconstruction relies on a tissue expander/submuscular or prepectoral plane insertion with the following exchange for a permanent implant. In contrast, a permanent implant is placed immediately after mastectomy during a single-stage reconstruction, known also as direct-to-implant procedures [22]. Improvement of surgery results and performance efficiency and reduction of reoperations rate can be obtained by integrating preoperative quantitative tissue assessment with five critical decisions in breast augmentation surgery [21]. Consequently, while making preoperative decisions, surgeons must consider five crucial areas of planning breast augmentation surgery. They are ordered by importance and includes optimal soft tissue coverage/breast implant pocket position, implant volume (weight), type, size and dimensions of the breast implant, proper location for the inframammary fold, and the incision place [21]. The following element of preoperative planning includes patient education [18,23]. A complete understanding of the patient’s entire breast augmentation process, choice of implant’s type, and shape are crucial aspects that should be clarified before intervention. The educational features should raise the patient’s awareness of the existing limitations of a procedure and the possibility of complications, along with the specific plan for the treatment after breast implantation.

## 7. Incision Site and Implant Placement

The incision before BI may be located in the inframammary, periareolar and transaxillary area [24]. Its selection depends on the patient’s individual preferences and potential benefits and risk factors [25]. Inframammary and periareolar incisions are the most popular choices [18,24,25]. The inframammary technique provides easy access, greater control through the precision of dissection and hemostasis of the pocket. Additionally, almost any type and size of breast implant might be used and damaging the breast tissue could be avoided. However, this type of incision is a potential risk factor of an unaesthetic scar formation. The periareolar incision allows proper access to the breast, and the formed scar mostly remains invisible. Nevertheless, poor scar formation, the higher risk of capsular contracture or an enhancement of the sensitivity of the nipple-areola complex are the potential complications. The undeniable advantage of the transaxillary incision is the lack of any scar in the breast area. Although, this method includes less control over the release of the pectoral muscle if an endoscope is not available and a lack of the possibility of dual-plane dissection. 

**Figure 4:**
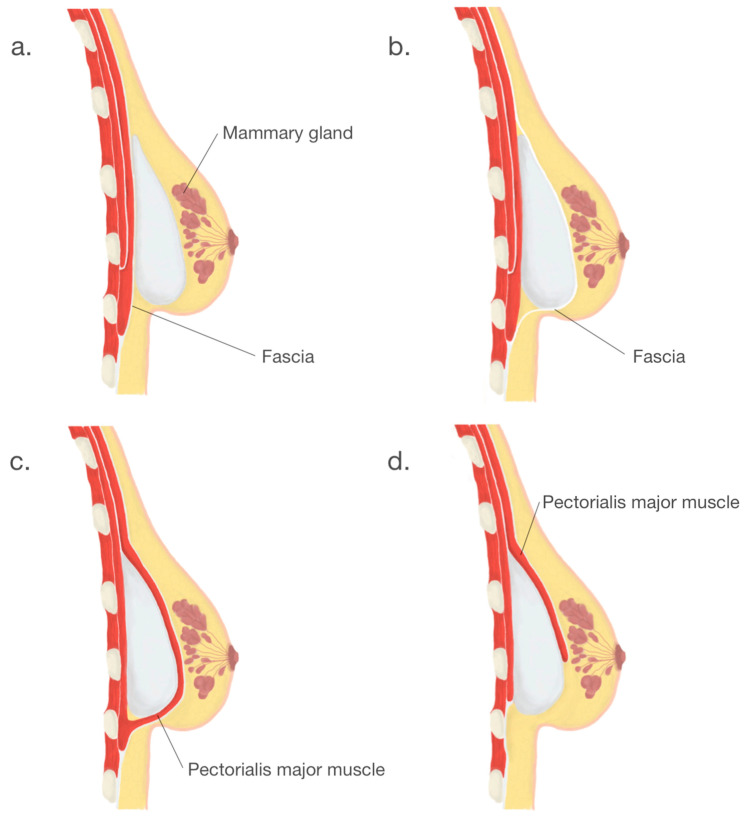
Breast implants placement locations: (**a**). subglandular, (**b**). subfascial, (**c**). submuscular, (**d**). dual—plane.

Another crucial step in BI surgery is implant placement. Usually, implants are placed either submuscularly—under the pectoral muscle or subglandularly—above the pectoral muscle but below the breast glandular parenchyma [26]. Submuscular placement of a breast implant is associated with a lower risk of capsule contracture and more accessible mammography imaging [5]. Due to the relative ease of surgery and the ability to achieve the desired cosmetic effect, the subglandular location remains a frequent choice. The following option is a subfascial placement consisting of placing the breast implant as an alternative to the submuscle location and represents a compromise between the submuscular and subglandular implantation [27]. Despite satisfactory results and rapidly gained popularity, the subfascial implant placement technique remains controversial. Another procedure, the dual-plane technique, remains a modification of the submuscle placement of the breast implant, which creates a plane of surgical dissection between the fascia of the pectoralis major and subglandular tissue muscle partially cover the surface of an implant [28]. According to the consensus of experts in breast augmentation from Australia and New Zealand, the dual-plane technique was the most frequently used [26]. Breast implants placement locations are shown in Figure 4.

## 8. Implant-Related Complications

Two common and acknowledged complications of breast augmentation surgery are implant rupture and capsular contracture [29]. The mechanisms behind the rupture of breast implants have been thoroughly investigated.25 Damage of the implant shell by surgical instruments, a flaw of the fold, swelling of the implant surface or a manufacturing defect are among the most common [30,31,32]. Risk factors for rupture of breast implants also include excessive forces on the chest, e.g., during closed capsulotomy, due to an injury with seat belts or blunt trauma, and the result of breast compression after mammography or severe capsular contracture. American manufacturers of breast implants regularly analyze the leading causes of ruptures of implants. Handel et al. analyzed data from Mentor and Allergan about breast implant fractures and concluded that approximately 51–64% of the implants were recorded as damaged by surgical instruments [31]. Breast implant ruptures are classified as intra-capsular and extra-capsular [33]. Intra-capsular rupture of a breast implant is complicated to identify with routine imaging methods such as mammography or ultrasound [34]. Therefore, it is usually detected during surgery.

Breast implant fractures can also be divided into overt and more frequent silent fractures [31]. Overt rupture of the breast implant is clinically visible, while additional imaging methods can only detect silent rupture. Therefore, this complication may go unnoticed by the patient and the physician [29,31]. The sensitivity of diagnosing a rupture of a breast implant is approximately 30% [31]. Various imaging techniques such as magnetic resonance imaging, mammography, ultrasound and computed tomography can diagnose breast implant rupture [35]. However, magnetic resonance imaging (MRI) is considered the standard of imaging as an excellent method for free silicone imaging and assessment of a breast implant rupture [36], with a sensitivity and specificity greater than 90%. In the case of contracture of the implant capsule, the formation of fibrosis around the implant remains a normal body response as an inserted breast implant acts as a foreign body [5].

The capsular contracture formation leads to the proliferation and differentiation of fibroblasts, ultimately leading to excessive collagen deposition [37]. Other factors causing capsule contraction include hematomas formed during surgery, periprosthetic infections, inflammatory cells’ chronic presence [38], along with inadequate pocket size for implant insertion and silicone leakage through the semipermeable shell of the breast implant.

Despite extensive research, the mechanism behind the contracture of the implant capsule remains unclear and not fully understood [38,39]. Bachour et al. suggest that the incidence of capsular contracture increases due to the gel leakage during implant rupture [40]. The longer the time from implant placement, the greater the cumulative risk of developing contracture of the implant capsule, which suggests a direct relationship between implant placement and the time to contracture [41]. In the first twelve months after surgery, 92% of the implant capsule contractures occur. This phenomenon is more common among breast reconstruction surgery patients due to pre- and postoperative exposure to chemotherapy and/or radiation therapy [41].

A greater risk of contracture of the implant capsule is associated with inserting a breast implant with a smooth surface and subglandular positioning. Various scales have been proposed to classify a grade of contracture of the breast implant capsule, e.g., Baker and Wilflingseder classifications [42], as shown in Table 1.

Notably, according to the Baker classification, only grade 3 and 4 of breast implant capsular contracture qualifies the patient for surgery [41,43]. Visible deformation of the implant, palpable hardness and pain are the main clinical manifestations of the contracture of the implant capsule. Each of these symptoms, especially the presence of pain, requires surgical intervention. Noteworthy, the Baker classification has been recently considered an unreliable diagnostic tool [44].

## 9. Breast Implant-Associated Anaplastic Large Cell Lymphoma

Breast implant-associated anaplastic large cell lymphoma (BIA-ALCL) is an uncommon non-Hodgkin’s T-cell lymphoma [45,46], characterized by a monoclonal population of CD30+ large anaplastic cells, negative anaplastic lymphoma kinase and variable expression of lymphocyte T and EMA markers [28,47]. The first case of BIA-ALCL described in 1997 by Keech and Creech was reported in a patient with saline implants [48]. In 2016, the World Health Organization acknowledged anaplastic large cell lymphoma (ALCL) associated with breast implants as a new type of malignancy [49,50], whereas In 2017, BIA-ALCL was included in the classification of haemato-lymphoid neoplasms [50]. Until now, the American Society of Plastic Surgeons Patient Registry has already collected 518 cases of BIA-ALCL from 25 countries [46], with the incidence defined at approximately one to three per million per year [46,51]. Nevertheless, the incidence of this condition is more prevalent than expected, as shown in recent Review by Santanelli di Pompeo et al. [50] Although most cases are indolent, the clinical features may include redness, breast pain and swelling, asymmetry, palpable tumour, seroma or ulceration [52,53,54]. Diagnosis is verified by cytological, immunohistochemical and immunophenotypic assessment of the aspirated peri-implant fluid [54]. Reports indicate that the ALCL affects patients with silicone and saline breast implants with a textured surface or tissue expanders [24,46,55]. Nearly all cases of ALCL are associated with textured surface breast implants [24,52,56]. However, this data was not confirmed in a meta-analysis conducted by Ramos-Gallardo et al. [28]. An increasing number of studies suggest a multifactorial cause of the development of BIA-ALCL: bacterial component, implant surface texture, genetic factors, and mechanical friction [45,56,57]. The presence of bacteria may reflect an opportunistic infection. The larger the surface area of the implant shell, the greater the probability of bacterial growth [45,46,52]. Jones et al. divided the textured implants into macro- and microtextured by considering the morphology of the outer shell of the textured implants [46]. They established a four-grade classification, with studies showing that the risk of developing ALCL is significantly higher for grades 3 and 4. 

Research into the genetic factors predisposing to the development of a BIA-ALCL is still ongoing, emphasising the activation of the JAK/STAT path among patients with confirmed lymphoma [52,58]. Total en-bloc capsulectomy is recommended in symptomatic patients as usually curative treatment [53,54,59]. However, late diagnosis may indicate a necessity of more invasive therapy [54]. Adjuvant chemotherapy, based on anthracycline with brentuximab vedotin following the ECHELON II trial results, is dedicated to patients with advanced disease [60,61]. As indicated by Rubio et al. if the implat removal was performed with no residual disease, the further follow-up requires a CT or a PET/CT scan assessment every six months for two years depending e patient’s clinical picture [62].

## 10. Breast Implant Illness

When the BI gained immense popularity, controversy regarding augmentation has also started to emerge [63,64]. The complications after surgery, such as the contracture of the implant capsule, rupture of the implant or its incorrect position, and possible relationships of inserted implants with autoimmune diseases, started to raise concerns. Currently, there are no studies that could confirm this theory [63,65,66]. However, a constantly increasing percentage of patients report symptoms negatively affecting their mental and physical functioning [54]. Chronic pain and fatigue, memory and concentration disorders, panic attacks, and depression [64]. Symptoms of Breast Implant Illness (BII) also include joints and muscles pain, mouth and eyes dryness, alopecia, skin lesions and Raynaud’s syndrome [65]. A significant group of patients manifesting these symptoms initiated linking them with breast implants, which eventually led to the creation of the BII term [11]. The history of this phrase dates back to the 1960s, when the compilation of symptoms occurring among patients for the first time was defined as a human adjuvant disease [11]. The term was then used to describe a potential relationship between injecting paraffin, petroleum products and silicone to patients and developing autoimmune connective tissue disorders. 

Currently, the term BII is used to describe a diverse and complex group of over 100 symptoms, focused on the following systems: the central nervous system, the musculoskeletal system, the immune system, the urogenital system, circulatory-respiratory system, the skin and its appendages and the psychological sphere [63,67]. The mechanism of development of BII is still unknown, even though research on possible associations of silicone breast implants with systemic diseases has been ongoing for over 40 years [66,68]. One of the theories explaining the occurrence of these non-specific symptoms is the development of an inflammatory or autoimmune reaction induced by the presence of a stimulant, in this case, silicone contained in breast implants [65]. Other theories suggest the pathophysiological basis of BII within social and psychological factors [63]. Unfortunately, the number of studies on BII is very limited. Currently, there are no diagnostic tests that would facilitate an adequate diagnosis, and in addition, there are no recommendations that would differentiate BII from other diseases [63,66]. Furthermore, BII is currently not considered a disease and has not been included by the World Health Organization in the latest ICD-11 classification. Nonetheless, Rohrich et al. indicate even more specific term for the syndrome called “silicone implant illness” [69]. In contrary, Lee et al. suggest BII symptoms seem not to correlate with any particular implant type, surface, or filling [67].

## 11. Breast Implant Removal

Decision considering breast implant removal results from various ailments, including those characterized as BII [55,70,71]. Scientific researches suggest that the longer the implant surgery, the greater the risk of cumulative complications such as contractures [72]. It indicates a direct correlation between implant insertion and the time of symptoms occurrence, affecting the frequency of implant removal. The patient should consciously make the decision about undergoing this procedure and be supported by up-to-date scientific evidence [73]. While planning further steps, it is essential to consider both the benefits and the risks of performing implant removal [54]. Proper informing the patient about the potential complications, such as the high frequency of physical defects, belongs to the duties of the attending physician [73]. Unfortunately, there is no guarantee that the BII symptoms will disappear after removing the implants, and the patient’s condition will significantly improve [55]. Although, it has been proven that the symptoms among patients without autoimmune disease are alleviated [55]. Consequently, patients must be informed that various degrees of capsulectomy/capsulotomy is widely recommended [70,71,73]. Defining a proper nomenclature presents some difficulties as inaccuracies exist even among plastic surgeons [73]. In en bloc removal surgery, the operator leaves the capsule tissue intact and then removes both the implant and the surrounding capsule as one unit with the breast tissue margin [70,73,74]. Most surgeons remove en bloc implants by inframammary fold incision due to the periareolar and transaxillary accesses visual limitations [73]. Total capsulectomy involves removal of the implant along with the capsule tissue surrounding the implant [74]. Meanwhile, partial capsulectomy involves implant removal and limited capsule tissue dissection [73]. Lastly, open capsulotomy involves the surgical release of a capsule or scar tissue surrounding the implant by dissecting the tissue with electrocautery in-vivo [73,74]. Misconduct in the state-of-art is an en bloc capsule removal, even if the pathology of the capsule cannot be proven [55]. The patients’ conviction about the necessity of an en bloc resection is motivated by the fear that an incompletely removed implant may produce toxic substances along with an increased risk of wound infection [73]. Nonetheless, the en-bloc technique is necessary for a malignant lesion, including ALCL [54,70]. However, no scientific basis suggests the need or benefit of using this technique to remove the benign capsule. Submuscular position and thin capsule increase the risk of significant bleeding and lead to a pneumothorax development during en bloc resection. The risk of developing a pneumothorax during en-bloc capsulectomy is approximately 4% [68]. Therefore, en-bloc capsulectomy should only be reserved for ALCL patients [75].

## 12. Authors Perspective

Implant-based breast reconstruction surgery is the most common method of breast reconstruction performed simultaneously with resection or as a deferred procedure. Obtaining a favourable aesthetic effect can be achieved by the correct selection of the size of the implant and careful surgical technique. Diverse scopes of implants, their texture and shape facilitate the selection of appropriate inserts. Implantation technique: prepectoral or retropectoral, synthetic meshes, or ADM depends on the anatomical conditions of the patient and the surgeon’s and patient’s preferences. The common use of implants in aesthetic or reconstructive breast surgery is reflected in the multitude of scientific papers available in scientific databases on the aspects of surgical technique, complications, the safety of their use or the results obtained.

## 13. Conclusions

The number of performed breast reconstructions is increasing, providing the finest aesthetic results and improving patient’s quality of life. Choice of prosthesis varies according to individual preferences and anatomical variables. A newly diagnosed cases of BIA-ALCL with lacking data on prevention, diagnosis, and treatment are placing it as a compelling medical challenge. Similarly, BII remains one of the most controversial subjects in reconstructive breast surgery due to unspecified diagnostic procedures, and recommendations.

## Figures and Tables

**Table 1:** Clinical classification (Baker score) and histological classification (Wilflingseder score) of capsular contracture.

Grade	Baker	Wilflingseder
I	Implant shell not palpable and not visible	Thin and uncontracted capsule
II	Implant shell slightly firm, but not visible	“Constrictive fibrosis”, no giant cells
III	Implant shell clearly firm and implant visible	“Constrictive fibrosis”, giant cells present
IV	Implant shell very firm, implant dislocation and deformation	Inflammatory cells, foreign body granulomas, neovascularization, possible neuromas

## Data Availability

Not applicable.

## References

[1] ISAPS International Survey on Aesthetic/Cosmetic Procedures Performed in 2019. https://www.isaps.org/medical-professionals/isaps-global-statistics/.

[2] National Plastic Surgery Statistics. https://www.plasticsurgery.org/news/plastic-surgery-statistics.

[3] Patel B.C., Wong C.S., Wright T., Schaffner A.D. (2021). Breast Implants.

[4] Song W.J., Kang S.G., Seo B.F., Choi N.K., Lee J.H. (2020). A Systematic Review of the National Breast Implant Registry for Application in Korea: Can We Predict “Unpredictable” Complications?. Medicina (Kaunas).

[5] Steinbach B.G., Hardt N.S., Abbitt P.L., Lanier L., Caffee H.H. (1993). Breast implants, common complications, and concurrent breast disease. Radiographics.

[6] Maxwell G.P., Gabriel A. (2014). The evolution of breast implants. Plast. Reconstr. Surg..

[7] Maxwell G.P., Gabriel A. (2017). Breast implant design. Gland Surg..

[8] Deva A.K., Cuss A., Magnusson M., Cooter R. (2019). The “Game of Implants”: A Perspective on the Crisis-Prone History of Breast Implants. Aesthet. Surg. J..

[9] Jewell M.L. (2012). Silicone gel breast implants at 50: The state of the science. Aesthet. Surg. J..

[10] Calobrace M.B., Capizzi P.J. (2014). The biology and evolution of cohesive gel and shaped implants. Plast. Reconstr. Surg..

[11] Magnusson M.R., Cooter R.D., Rakhorst H., McGuire P.A., Adams W.P., Deva A.K. (2019). Breast Implant Illness: A Way Forward. Plas.t Reconstr. Surg..

[12] Calobrace M.B., Schwartz M.R., Zeidler K.R., Pittman T.A., Cohen R., Stevens W.G. (2017). Long-Term Safety of Textured and Smooth Breast Implants. Aesthet. Surg. J..

[13] Cole N.M. (2018). Consequences of the U.S. Food and Drug Administration-Directed Moratorium on Silicone Gel Breast Implants: 1992 to 2006. Plast. Reconstr. Surg..

[14] McCarthy C.M., Cano S.J., Klassen A.F., Scott A., Van Laeken N., Lennox P.A., Cordeiro P.G., Pusic A.L. (2012). The magnitude of effect of cosmetic breast augmentation on patient satisfaction and health-related quality of life. Plast. Reconstr. Surg..

[15] https://www.fda.gov/media/80685/download.

[16] Shridharani S.M., Bellamy J.L., Mofid M.M., Singh N.K. (2013). Breast augmentation. Eplasty.

[17] Rocco N., Rispoli C., Moja L., Amato B., Iannone L., Testa S., Spano A., Catanuto G., Accurso A., Nava M.B. (2016). Different types of implants for reconstructive breast surgery. Cochrane Database Syst. Rev..

[18] Adams W.P., Mallucci P. (2012). Breast augmentation. Plast. Reconstr. Surg..

[19] Hidalgo D.A., Spector J.A. (2014). Breast augmentation. Plast. Reconstr. Surg..

[20] Colwell A.S., Taylor E.M. (2020). Recent Advances in Implant-Based Breast Reconstruction. Plast. Reconstr. Surg..

[21] Tebbetts J.B., Adams W.P. (2006). Five critical decisions in breast augmentation using five measurements in 5 minutes: The high five decision support process. Plast. Reconstr. Surg..

[22] Frey J.D., Salibian A.A., Karp N.S., Choi M. (2019). Implant-Based Breast Reconstruction: Hot Topics, Controversies, and New Directions. Plast. Reconstr. Surg..

[23] Brown M.H., Somogyi R.B., Aggarwal S. (2016). Secondary Breast Augmentation. Plast. Reconstr. Surg..

[24] Schwartz M.R. (2017). Evidence-Based Medicine: Breast Augmentation. Plast. Reconstr. Surg..

[25] Goes J.C., Landecker A. (2003). Optimizing outcomes in breast augmentation: Seven years of experience with the subfascial plane. Aesthet. Plast. Surg..

[26] Benito-Ruiz J., Redondo A. (2020). Breast Augmentation Surgery: How Do We Do It? Results of a Joint Survey from European Association of Societies of Aesthetic Plastic Surgery. Aesthet. Plast. Surg..

[27] Atiyeh B.S., Chahine F. (2021). Comment on A Comprehensive Outcome Review of Subfascial Breast Augmentation over a 10-Year Period. Aesthet. Plast. Surg..

[28] Ramos-Gallardo G., Cuenca-Pardo J., Rodriguez-Olivares E., Iribarren-Moreno R., Contreras-Bulnes L., Vallarta-Rodriguez A., Kalixto-Sanchez M., Hernandez C., Ceja-Martinez R., Torres-Rivero C. (2017). Breast Implant and Anaplastic Large Cell Lymphoma Meta-Analysis. J. Investig. Surg..

[29] Holmich L.R., Vejborg I.M., Conrad C., Sletting S., Hoier-Madsen M., Fryzek J.P., McLaughlin J.K., Kjoller K., Wiik A., Friis S. (2004). Untreated silicone breast implant rupture. Plast. Reconstr. Surg..

[30] Hillard C., Fowler J.D., Barta R., Cunningham B. (2017). Silicone breast implant rupture: A review. Gland Surg..

[31] Handel N., Garcia M.E., Wixtrom R. (2013). Breast implant rupture: Causes, incidence, clinical impact, and management. Plast. Reconstr. Surg..

[32] Necchi S., Molina D., Turri S., Rossetto F., Rietjens M., Pennati G. (2011). Failure of silicone gel breast implants: Is the mechanical weakening due to shell swelling a significant cause of prostheses rupture?. J. Mech. Behav. Biomed. Mater..

[33] Pinchuk V., Tymofii O., Tkach O., Zamkovoy V. (2013). Implant ruptures after augmentation mammoplasty. Aesthet. Plast. Surg..

[34] Rukanskiene D., Bytautaite G., Cesnauskaite A., Pilipaityte L., Astrauskas T., Jonaitiene E. (2021). The Value of Ultrasound in the Evaluation of the Integrity of Silicone Breast Implants. Medicina (Kaunas).

[35] Gorczyca D.P., Gorczyca S.M., Gorczyca K.L. (2007). The diagnosis of silicone breast implant rupture. Plast. Reconstr. Surg..

[36] Lin M.-F., Lai L.-H., Hsiao W.-T., Yao M.M.-S., Chan W.-P. (2021). Developing a Specific MRI Technology to Identify Complications Caused by Breast Implants. Appl. Sci..

[37] Diehm Y.F., Jost Y., Kotsougiani-Fischer D., Haug V., Splinter M., Haring P., Berger M.R., Debus J., Kneser U., Fischer S. (2021). The Treatment of Capsular Contracture Around Breast Implants Induced by Fractionated Irradiation: The Collagenase of the Bacterium Clostridium Histolyticum as a Novel Therapeutic Approach. Aesthet. Plast. Surg..

[38] Gundeslioglu O., Altundag O., Altundag K. (2005). Nanobacteria and breast implant capsule contracture and calcification: A hypothesis. Aesthet. Plast. Surg..

[39] Bachour Y. (2021). Capsular Contracture in Breast Implant Surgery: Where Are We Now and Where Are We Going?. Aesthet. Plast. Surg..

[40] Bachour Y., Bargon C.A., de Blok C.J.M., Ket J.C.F., Ritt M., Niessen F.B. (2018). Risk factors for developing capsular contracture in women after breast implant surgery: A systematic review of the literature. J. Plast. Reconstr. Aesthet. Surg..

[41] Araco A., Caruso R., Araco F., Overton J., Gravante G. (2009). Capsular contractures: A systematic review. Plast. Reconstr. Surg..

[42] Prantl L., Schreml S., Fichtner-Feigl S., Poppl N., Eisenmann-Klein M., Schwarze H., Fuchtmeier B. (2007). Clinical and morphological conditions in capsular contracture formed around silicone breast implants. Plast. Reconstr. Surg..

[43] Prantl L., Shiffman M.A. (2009). Serologic and Histologic Findings in Capsule Contracture Patients with Silicone Gel Implants. Breast Augmentation: Principles and Practice.

[44] de Bakker E., Rots M., Buncamper M.E., Niessen F.B., Smit J.M., Winters H.A.H., Ozer M., de Vet H.C.W., Mullender M.G. (2020). The Baker Classification for Capsular Contracture in Breast Implant Surgery Is Unreliable as a Diagnostic Tool. Plast. Reconstr. Surg..

[45] Groth A.K., Graf R. (2020). Breast Implant-Associated Anaplastic Large Cell Lymphoma (BIA-ALCL) and the Textured Breast Implant Crisis. Aesthet. Plast. Surg..

[46] Jones J.L., Hanby A.M., Wells C., Calaminici M., Johnson L., Turton P., Deb R., Provenzano E., Shaaban A., Ellis I.O. (2019). Breast implant-associated anaplastic large cell lymphoma (BIA-ALCL): An overview of presentation and pathogenesis and guidelines for pathological diagnosis and management. Histopathology.

[47] Rondon-Lagos M., Rangel N., Camargo-Villalba G., Forero-Castro M. (2021). Biological and genetic landscape of breast implant-associated anaplastic large cell lymphoma (BIA-ALCL). Eur. J. Surg. Oncol..

[48] Keech J.A., Creech B.J. (1997). Anaplastic T-cell lymphoma in proximity to a saline-filled breast implant. Plast. Reconstr. Surg..

[49] Swerdlow S.H., Campo E., Pileri S.A., Harris N.L., Stein H., Siebert R., Advani R., Ghielmini M., Salles G.A., Zelenetz A.D. (2016). The 2016 revision of the World Health Organization classification of lymphoid neoplasms. Blood.

[50] di Pompeo S.F., Sorotos M., Clemens M.W., Firmani G., European Association of Plastic Surgeons (EURAPS) Committee on Device Safety and Development (2021). Breast Implant-Associated Anaplastic Large Cell Lymphoma (BIA-ALCL): Review of Epidemiology and Prevalence Assessment in Europe. Aesthet. Surg. J..

[51] Nava M.B., Adams W.P., Botti G., Campanale A., Catanuto G., Clemens M.W., Del Vecchio D.A., De Vita R., Di Napoli A., Hall-Findlay E. (2018). MBN 2016 Aesthetic Breast Meeting BIA-ALCL Consensus Conference Report. Plast. Reconstr. Surg..

[52] Cuomo R. (2021). The State of the Art about Etiopathogenetic Models on Breast Implant Associated-Anaplastic Large Cell Lymphoma (BIA-ALCL): A Narrative Review. J. Clin. Med..

[53] Clemens M.W., Brody G.S., Mahabir R.C., Miranda R.N. (2018). How to Diagnose and Treat Breast Implant-Associated Anaplastic Large Cell Lymphoma. Plast. Reconstr. Surg..

[54] Turton P., El-Sharkawi D., Lyburn I., Sharma B., Mahalingam P., Turner S.D., MacNeill F., Johnson L., Hamilton S., Burton C. (2021). UK Guidelines on the Diagnosis and Treatment of Breast Implant-Associated Anaplastic Large Cell Lymphoma on behalf of the Medicines and Healthcare products Regulatory Agency Plastic, Reconstructive and Aesthetic Surgery Expert Advisory Group. Br. J. Haematol..

[55] McGuire P.A., Haws M.J., Nahai F. (2019). Breast Implant Illness: How Can We Help?. Aesthet. Surg. J..

[56] Deva A.K., Turner S.D., Kadin M.E., Magnusson M.R., Prince H.M., Miranda R.N., Inghirami G.G., Adams W.P. (2020). Etiology of Breast Implant-Associated Anaplastic Large Cell Lymphoma (BIA-ALCL): Current Directions in Research. Cancers.

[57] Mempin M., Hu H., Chowdhury D., Deva A., Vickery K. (2018). The A, B and C’s of Silicone Breast Implants: Anaplastic Large Cell Lymphoma, Biofilm and Capsular Contracture. Materials.

[58] Oishi N., Miranda R.N., Feldman A.L. (2019). Genetics of Breast Implant-Associated Anaplastic Large Cell Lymphoma (BIA-ALCL). Aesthet. Surg. J..

[59] Clemens M.W., Jacobsen E.D., Horwitz S.M. (2019). 2019 NCCN Consensus Guidelines on the Diagnosis and Treatment of Breast Implant-Associated Anaplastic Large Cell Lymphoma (BIA-ALCL). Aesthet. Surg. J..

[60] Duvic M., Tetzlaff M.T., Gangar P., Clos A.L., Sui D., Talpur R. (2015). Results of a Phase II Trial of Brentuximab Vedotin for CD30+ Cutaneous T-Cell Lymphoma and Lymphomatoid Papulosis. J. Clin. Oncol..

[61] Prince H.M., Kim Y.H., Horwitz S.M., Dummer R., Scarisbrick J., Quaglino P., Zinzani P.L., Wolter P., Sanches J.A., Ortiz-Romero P.L. (2017). Brentuximab vedotin or physician’s choice in CD30-positive cutaneous T-cell lymphoma (ALCANZA): An international, open-label, randomised, phase 3, multicentre trial. Lancet.

[62] Sanchez Rubio N., Lannegrand Menendez B., Duque Munoz M., Montes Fernandez M., Ciudad Fernandez M.J. (2020). Uncommon complications of breast prostheses. Radiologia (Engl. Ed.).

[63] Newby J.M., Tang S., Faasse K., Sharrock M.J., Adams W.P. (2020). Understanding Breast Implant Illness. Aesthet. Surg. J..

[64] Atiyeh B., Emsieh S. (2021). Breast Implant Illness (BII): Real Syndrome or a Social Media Phenomenon? A Narrative Review of the Literature. Aesthet. Plast. Surg..

[65] Wee C.E., Younis J., Isbester K., Smith A., Wangler B., Sarode A.L., Patil N., Grunzweig K., Boas S., Harvey D.J. (2020). Understanding Breast Implant Illness, Before and After Explantation: A Patient-Reported Outcomes Study. Ann. Plast. Surg..

[66] Tang S.Y.Q., Israel J.S., Afifi A.M. (2017). Breast Implant Illness: Symptoms, Patient Concerns, and the Power of Social Media. Plast. Reconstr. Surg..

[67] Lee M., Ponraja G., McLeod K., Chong S. (2020). Breast Implant Illness: A Biofilm Hypothesis. Plast. Reconstr. Surg. Glob. Open.

[68] Keane G., Chi D., Ha A.Y., Myckatyn T.M. (2021). En Bloc Capsulectomy for Breast Implant Illness: A Social Media Phenomenon?. Aesthet. Surg. J..

[69] Rohrich R.J., Kaplan J., Dayan E. (2019). Silicone Implant Illness: Science versus Myth?. Plast. Reconstr. Surg..

[70] Swanson E. (2020). Evaluating the Necessity of Capsulectomy in Cases of Textured Breast Implant Replacement. Ann. Plast. Surg..

[71] Swanson E. (2021). The Case for Breast Implant Removal or Replacement Without Capsulectomy. Aesthet. Plast. Surg..

[72] Gurunluoglu R., Sacak B., Arton J. (2013). Outcomes analysis of patients undergoing autoaugmentation after breast implant removal. Plast. Reconstr. Surg..

[73] Kaplan J., Rohrich R. (2021). Breast implant illness: A topic in review. Gland Surg..

[74] Gerzenshtein J. (2020). The Dishonesty of Referring to Total Intact Capsulectomy as “En Bloc” Resection or Capsulectomy. Plast. Reconstr. Surg..

[75] Tevis S.E., Hunt K.K., Clemens M.W. (2019). Stepwise En Bloc Resection of Breast Implant-Associated Anaplastic Large Cell Lymphoma with Oncologic Considerations. Aesthet. Surg. J. Open Forum..

